# The mediating role of shame in the relationship between childhood bullying victimization and adult psychosocial adjustment

**DOI:** 10.1080/20008198.2017.1418570

**Published:** 2018-01-16

**Authors:** Ida Frugård Strøm, Helene Flood Aakvaag, Marianne Skogbrott Birkeland, Erika Felix, Siri Thoresen

**Affiliations:** ^a^ Norwegian Centre for Violence and Traumatic Stress Studies, Oslo, Norway; ^b^ Department of Counseling, Clinical, & School Psychology, University of California, Santa Barbara, Santa Barbara, CA, USA

**Keywords:** Bullying, violence, shame, psychosocial adjustment, young adulthood, bullying o acoso, violencia, vergüenza, ajuste psicosocial, edad adulta, 霸凌, 暴力, 羞耻, 心理社会适应, 年轻成人, • Bullying victimization is often studied in isolation from other forms of victimization.• Childhood bullying victimization had a unique effect, over and above the effect of violence, on psychosocial adjustment (psychological distress, impaired functioning, social support barriers) in young adults who experienced bullying.• Shame explained a large part of the association between experiencing bullying victimization and psychosocial adjustment in young adulthood.• Bullying victimization should be included and integrated in trauma research to a larger extent, along with other forms of violence.

## Abstract

**Background**: Psychological distress following experiencing bullying victimization in childhood has been well documented. Less is known about the impact of bullying victimization on psychosocial adjustment problems in young adulthood and about potential pathways, such as shame. Moreover, bullying victimization is often studied in isolation from other forms of victimization.

**Objective**: This study investigated (1) whether childhood experiences of bullying victimization and violence were associated with psychosocial adjustment (distress, impaired functioning, social support barriers) in young adulthood; (2) the unique effect of bullying victimization on psychosocial adjustment; and (3) whether shame mediated the relationship between bullying victimization and these outcomes in young adulthood.

**Method**: The sample included 681 respondents (aged 19–37 years) from a follow-up study (2017) conducted via phone interviews derived from a community telephone survey collected in 2013.

**Results**: The regression analyses showed that both bullying victimization and severe violence were significantly and independently associated with psychological distress, impaired functioning, and increased barriers to social support in young adulthood. Moreover, causal mediation analyses indicated that when childhood physical violence, sexual abuse, and sociodemographic factors were controlled, shame mediated 70% of the association between bullying victimization and psychological distress, 55% of the association between bullying victimization and impaired functioning, and 40% of the association between bullying victimization and social support barriers.

**Conclusions**: Our findings support the growing literature acknowledging bullying victimization as a trauma with severe and long-lasting consequences and indicate that shame may be an important pathway to continue to explore. The unique effect of bullying victimization, over and above the effect of violence, supports the call to integrate the two research fields.

## Abstract (es)

**Planteamiento:** La angustia psicológica después de haber experimentado victimización por acoso en la infancia ha sido bien documentada. Se sabe menos sobre el impacto de la victimización por acoso en los problemas de ajuste psicosocial en la adultez joven y sobre vías potenciales, como la vergüenza. Además, la victimización por acoso a menudo se estudia aisladamente de otras formas de victimización.

**Objetivo:** Este estudio investigó (1) si las experiencias infantiles de victimización por acoso y violencia estaban asociadas con el ajuste psicosocial (angustia, funcionamiento, barreras al apoyo social) en la edad adulta; (2) el efecto único de la victimización por intimidación en el ajuste psicosocial; y (3) si la vergüenza mediaba en la relación entre la victimización por acoso y estos resultados en la adultez temprana.

**Método:** La muestra incluyó 681 encuestados (con edades entre 19 y 37 años) de un estudio de seguimiento (2017) realizado a través de entrevistas telefónicas derivadas de una encuesta telefónica comunitaria recopilada en 2013. Resultados: Los análisis de regresión mostraron que tanto la victimización por acoso como la violencia grave se asociaban de manera significativa e independiente con la angustia psicológica, el deterioro del funcionamiento y el aumento de las barreras al apoyo social en la edad adulta. Además, los análisis de mediación causal indicaron que cuando se controlaba la violencia física infantil, el abuso sexual y los factores sociodemográficos, la vergüenza mediaba en un 70% de la asociación entre la victimización por acoso y la angustia psicológica, en un 55% de la asociación entre la victimización por acoso y el deterioro del funcionamiento y en el 40% de la asociación entre la victimización por acoso y las barreras al apoyo social.

**Conclusiones:** Nuestros hallazgos apoyan la creciente literatura que reconoce la victimización por acoso como un trauma con consecuencias severas y duraderas, e indican que la vergüenza puede ser un camino importante para continuar explorando. El efecto singular de la victimización por acoso, más allá del efecto de la violencia, respalda la llamada a integrar los dos campos de investigación.

## Abstract (zh)

**背景：**童年期经历霸凌后的心理痛苦早就被记录。但关于霸凌受害对成年早期心理社会适应问题的影响，以及可能路径（比如羞耻）还了解较少。此外，霸凌受害通常和其它的受害形式分开研究。

**目标：**本研究考察了：(1）童年霸凌受害和暴力经验是否和心理社会适应（痛苦感、功能、社会支持障碍）相关；(2）霸凌受害对心理社会适应的独特效应；(3）羞耻是否中介了霸凌受害和成年早期表现之间的关系。

**方法：**样本来自对2013年的一个社会电话调查进行的追踪电话采访研究（2017年），包括681名被试（年龄19-37岁）。

**结果：**回归分析显示霸凌书海和严重暴力都分别和心理痛苦、功能受损以及成年早期的社会支持障碍显著相关。此外，因果中介分析显示控制童年肢体暴力、性虐待和社会人口学因素后，羞耻分别中介了霸凌受害和心理痛苦之间关系的70%，和功能受损关系的55%，和社会支持障碍关系的40%。

**结论：**我们的发现支持了越来越多的文献认可霸凌受害是一类具有严重和长期后果的创伤，同时提示了羞耻可能是一条重要的有待探索的路径。霸凌受害表现的独特效应超过了暴力，支持了对结合两个领域的呼声。

## 1. Introduction

Bullying victimization is a common experience that affects the lives of a significant proportion of children and adolescents (Srabstein & Leventhal, 2010). Prevalence rates vary greatly across studies and countries, but a meta-analysis of 80 studies found a mean prevalence rate of 36% for bullying victimization (Modecki, Minchin, Harbaugh, Guerra, & Runions). Bullying has been defined as a subcategory of aggressive behaviour that is intended to harm or disturb, occurs repeatedly over time, involves an imbalance of power between the aggressor and target, and the victim has a difficult time stopping the victimization or defending him- or herself (Olweus, 1993). Bullying can be direct (i.e. an open attack) or indirect (i.e. social isolation and exclusion) and can be physical (e.g. hitting or pushing), verbal (e.g. name calling), or relational (with the intent to damage relationships; e.g. social exclusion or spreading rumours) (Furlong, Soliz, Simental, & Grief, 2004; Liu & Graves, 2011; Olweus, 1999). Thus, bullying victimization shares some central features with other types of victimization, such as child maltreatment (Krug, Dahlberg, Mercy, Zwi, & Lozano, 2002), that are commonly considered by violence researchers. However, bullying victimization and other forms of violence are often studied in isolation, being the focus of separate research traditions, although we know that they often co-occur (Espelage, Hong, & Mebane, 2016; Wolke, Copeland, Angold, & Costello, 2013). Finkelhor, Ormrod, and Turner (2007) argue that focusing on one specific form of childhood victimization in isolation may lead to an overestimation of its effects because of the large overlap among victimization experiences. Studying bullying victimization without taking violence into account may therefore bear the risk of overestimating its association with potential negative consequences.

### 1.1. Psychosocial adjustment following bullying victimization

The long-term negative physical (e.g. physical illness, poor general health) and mental health (e.g. anxiety, depression) consequences of bullying victimization have been well documented (Copeland, Wolke, Angold, & Costello, 2013; Takizawa, Maughan, & Arseneault, 2014; Ttofi & Farrington, 2012; Wolke et al., 2013). Some studies have even found this association to have an influence over and above other forms of childhood victimization (Espelage et al., 2016; Lereya, Copeland, Costello, & Wolke, 2015). Childhood violence researchers have begun to explore adverse effects beyond health, such as impaired functioning and social relationships, a development that has just begun within bullying research. The few studies that have investigated bullying victimization and broader aspects of psychosocial adjustment have shown that the consequences last well into adulthood in terms of impaired functioning, such as problems with doing housework and managing money (Laditka & Laditka, 2017), reduced levels of education (Sigurdson, Wallander, & Sund, 2014; Strøm et al., 2013), and problems with work (Sansone, Leung, & Wiederman, 2013; Strøm, 2014; Varhama & Björkqvist, 2005). These are important findings as we need to understand the impact of bullying on a diverse range of adult adjustment outcomes.

Bullying victimization may also influence social relationships. In childhood, victims of bullying are more likely to report loneliness, social avoidance, and self-blame (Arseneault, Bowes, & Shakoor, 2010; Graham & Juvonen, 1998; Olweus, 1993; Schacter, White, Chang, & Juvonen, 2015). They experience more rejection and feel less accepted by peers (Cullerton-Sen & Crick, 2005; Veenstra et al., 2007). Not surprisingly, they may have trouble forming new social relationships during the transition to young adulthood and experience more loneliness, less social support, a lower likelihood of having a live-in partner, and poorer family functioning in adulthood (Day et al., 2016; Sigurdson et al., 2014). Wolke et al. (2013) found that victims of bullying were at increased risk for problems with finances, health, and social relationships in adulthood, even when controlling for other adversity, including childhood violence.

Taken together, these findings show the unique effect of bullying victimization on a wide spectrum of psychosocial adjustment and indicate that it may have negative long-term consequences similar to those of childhood violence.

### 1.2. Shame as a mediator in the relationship between bullying victimization and psychosocial adjustment problems

Although bullying victimization seems to influence psychosocial adjustment (defined here as psychological distress, impaired functioning, and experiencing barriers to social support) in adulthood, the pathways for this relationship are not well understood. One possible pathway is the shame that may accompany victimization. Several empirical studies support that shame is a common response to interpersonal violence and to such highly stigmatized phenomena as sexual abuse (Amstadter & Vernon, 2008), and that shame is related to mental health (Aakvaag et al., 2016; Andrews, Brewin, Rose, Kirk, & Strauss, 2000; Beck et al., 2011; La Bash & Papa, 2014). The negative thoughts and feelings about the self (shame) that may follow a traumatic experience are also included in the symptom criterion for the PTSD diagnosis in the fifth edition of the DSM (American Psychiatric Association, 2013). Shame is a painful emotion that may reflect the victim’s belief that others in their social surroundings may judge them as having negative personal attributes and characteristics or as having engaged in negative behaviours (Gilbert, 2000). Though it is frequently observed that many victims of violence feel shameful about their experience, much remains unknown regarding why such feelings occur and how they affect mental health and other potential outcomes. Theorists who seek to explain the occurrence of shame in violence victims often underscore the social nature of shame. Shame may arise if the violent experience is perceived as an attack of the self, is associated with loss of status or social attractiveness, or involves acute domination or subjugation (Budden, 2009; Lee, Scragg, & Turner, 2001). The same theoretical framework could be applied to bullying victimization, as the experience of bullying may also be seen as a threat to the individual’s social self, may involve domination and subjugation, and may result in a loss of social status and attractiveness. Being excluded by your social group during adolescence, when one’s peers are of crucial importance and one’s social identity is developing, may make bullying victims especially vulnerable to shame. In addition, bullying victimization is often visible to others and can involve humiliation, two factors that are central to shame (Andrews et al., 2000; Wilson, Droždek, & Turkovic, 2006). Although shame has received increased attention in violence research, less is known about how bullying victimization relates to shame. To date, the bullying literature has mainly focused on the shame experienced by the bully or bystander, not shame experienced by the target of bullying (Ahmed & Braithwaite, 2004; Mazzone, Camodeca, & Salmivalli, 2016; Olthof, 2012; Olthof, Schouten, Kuiper, Stegge, & Jennekens-Schinkel, 2000). A recent study found that the victim’s shame mediated the association between experiencing peer victimization and a range of psychological distress in adolescents, suggesting that shame may be a mechanism by which peer victimization may influence mental health (Irwin, Li, Craig, & Hollenstein, 2016).

Shame can elicit withdrawal behaviour and social isolation, meaning that when individuals experience shame, they tend to pull back from their social relationships, thus potentially influencing their degree of social support (Nathanson, 1992; Wilson et al., 2006). Shame may also result from how the social group responds to the individual. Negative responses to being a victim of violence from their support network are unfortunately quite common and may cause survivors to feel shameful (Hershkowitz, Lanes, & Lamb, 2007; Ullman, 1999). In line with this, shame has been found to associate with negative expectations regarding social support, including the extent to which support is considered useful (Dodson & Beck, 2017). Thus, shame after bullying victimization may instigate barriers to social support seeking.

Behavioural aspects of shame, including social withdrawal, isolation, and rumination (Cheung, Gilbert, & Irons, 2004), may influence the management of school/work and relationships with friends and family. In addition, as shame is intensely painful, it may result in maladaptive avoidance strategies (Kim, Thibodeau, & Jorgensen, 2011), such as substance abuse or risk taking, which may have a further negative effect on psychosocial adjustment. However, research in this area is lacking.

### 1.3. Current study

In summary, there is a lack of knowledge regarding the unique effect of bullying victimization, over and above childhood violence exposure, on psychosocial adjustment in adulthood. In addition, the potential pathways leading to poor psychosocial adjustment must be examined to inform potential interventions to support victims of bullying. Thus, we sought to: (1) investigate whether childhood experiences of bullying victimization and violence are associated with mental health, impaired functioning, and social support barriers in young adulthood; (2) examine the unique effect of bullying victimization on these psychosocial adjustment outcomes; and (3) assess whether shame mediates the relationship between bullying victimization and these outcomes in young adulthood.

## 2. Method

### 2.1. Participants and procedure

This is the second follow-up study (T3) of a subsample of participants from a population study on violence exposure (T1) and a first follow-up study (T2). The population study (T1) was conducted in 2013 via phone interviews that included an adolescent sample (aged 16–17 years, *n* = 2062) and an adult sample (aged 18–75 years, *n* = 4527) drawn from the General Population Registry of Norway (see Thoresen, Myhre, Wentzel-Larsen, Aakvaag, & Hjemdal, 2015, for more details about the procedure at T1). Of these participants, 88.6% (*N* = 5838) consented to be contacted for follow-up studies. As we only wanted to include young adults at T2, the youngest individuals (aged 17–33 years at T1; *n* = 2549) were interviewed by phone 12–18 months after the baseline survey. We contacted respondents who were exposed to childhood violence (cases) and respondents who were not (controls). Of the individuals who answered the phone (*n* = 1224), 1010 (82.6%) participated, accounting for 39.7% of the individuals we attempted to reach. The final T2 sample thus included 506 cases and 504 controls. The T3 data were collected by phone during the last quarter of 2016 and the first quarter of 2017. We attempted to contact individuals from the T1 sample who were not reached at T2 (1875) and individuals from the T2 sample (*n* = 1003; *N* = 2878). Of these, we were unable to reach 1800 because of technical errors, no answer, incorrect registration information, incorrect numbers, and unattended follow-up appointments. Of the individuals who answered the phone (*n* = 1078), 63.2% (*n* = 681) participated (see Appendix). The majority of the participants were from the T2 sample (57.7%, *n* = 394) and the rest were from the T1 sample (42.3%, *n* = 287). The final sample included 42% cases (*n* = 286) and 58% controls (*n* = 395). All three studies were conducted by the data collection agency Ipsos and were approved by the Regional Committee for Medical and Health Research Ethics in southeast Norway.

#### 2.1.1. *Sample description (*N* = 681*)

The study sample consisted of a slight predominance of women (54.2%, *n* = 369). The age at baseline ranged from 16–33 years, with a mean age of 21 years (*SD*: 5.66); at T3, the age ranged from 19–37 years, with a mean age of 25 years (*SD*: 5.76). The majority of the sample had Norwegian-born parents (95.3%, *n* = 649). The self-reported financial situation at T1 were relatively high, with most of the sample reporting an income similar to (63.7%, *n* = 431) or better than that of most people in Norway (28.1%, *n* = 190).

## 3. Measures

### 3.1. T1 measures

#### 3.1.1. Childhood severe violence

Childhood severe violence was defined in this study as an affirmative response to any of the following: sexual abuse occurring before 13 years of age, forcible rape before age 18 years, and/or severe physical violence from parents before 18 years of age. *Forcible rape* included forceful intercourse, oral sex, anal sex, or having had fingers or objects put in the vagina or anus using physical force or threats. In addition to forcible rape, it is important to capture sexual abuse in younger kids. Adults or older teenagers can trick young kids into sexual behaviours without necessary using physical force or overt threats. The age limit of 13 is strict, but is meant to capture pre-pubertal sexual abuse. In addition, to exclude sexual play, we used the criteria that the perpetrator should be at least five years older. Our question on *sexual abuse occurring before 13 years of age* was introduced as follows: ‘Sometimes children can be tricked, rewarded or threatened to engage in sexual acts they don’t understand or are unable to stop’; followed by, ‘Before you were 13 years of age, did anyone who was at least 5 years older than you have any form of sexual contact with you?’ If the respondent answered affirmatively, follow-up questions asked if the sexual act included vaginal, oral, or anal penetration (Kilpatrick, Resnick, Baber, Guille, & Gros, 2011). *Severe physical violence from parents* included four forms of violence: having been (1) hit with a fist or a hard object, (2) kicked, (3) beaten up, and/or (4) physically attacked in other ways.

#### 3.1.2. Demographics

The information collected included age, gender, ethnicity, parents’ mental health problems, and perceived family financial situation. Parents’ mental health problems were measured with a single question adapted from the ACE study (Felitti et al., 1998) asking the respondents whether ‘a parent or other adults in your family of origin have mental health problems’, with the responses ‘yes’, ‘no’, and ‘don’t know/do not wish to answer’.

### 3.2. T3 measures

#### 3.2.1. Bullying victimization

The participants responded to questions about the three main components of bullying (chronicity, intentionality, and imbalance of power), derived from the California Bullying Victimization Scale (CBVS), a behavioural-based self-report questionnaire developed by Felix, Sharkey, Green, Furlong, and Tanigawa (2011). An adjusted retrospective version of the CBVS, which has shown good psychometric properties, was used in this study (Green et al., In Press). The respondents were first presented with the following information: ‘The following questions concern experiences in primary school and lower and upper secondary school. Did it EVER happen to you that someone on purpose in a mean or hurtful way … (1) teased or called you names? (2) spread rumours or gossip about you? (3) excluded you from a group or ignored you on purpose? (4) hit, pushed or physically injured you? (5) stole or damaged your things on purpose? (6) teased, had rumours spread about you, or threatened you through the Internet (like on a social network site or e-mail) on purpose by a student at your school?’ The respondents who answered yes to one or more items were then asked how many times each incidence had occurred when it was at its worst, with the response categories ‘rarely’, ‘multiple times a month or more’, ‘don’t know/do not wish to answer’. The respondents who answered ‘multiple times a month or more’ for one or more items were asked follow-up questions regarding whether they were able to defend themselves against the bully(ies) and his/her (their) actions, with the following response categories: Yes, definitely; Yes, to some extent; No, not usually; No, definitely not (Green et al., In Press). Only the respondents who answered affirmatively to the two latter categories were considered to have been bullied.

#### 3.2.2. Shame

Participants responded to the shame subscale of the Shame and Guilt after Trauma Scale (SGATS, authors’ own). The four items included were (1) Have you worried about what other people might think of you after what happened? (2) Have you tried to conceal what happened, or any part of it? (3) Have you felt ashamed about any part of what happened? (4) Have you looked down on yourself after what happened? The questions were posed to individuals who had experienced a violent or potentially traumatic event (e.g. life-threatening disease; death of a loved one due to accident, murder, or suicide; serious injury; or experiencing a terrifying situation), as the shame items sought to measure trauma-related shame. Individuals who had experienced multiple traumatic incidences (such as both bullying victimization and severe violence) were asked to base their response on the worst perceived event, which is a common strategy when asking about posttraumatic stress (Norris & Hamblen, 2004). Mean scores (ranging from 0–2) were calculated based on the three response categories: no, yes, a little, yes, a lot. Cronbach’s alpha was 0.84.

#### 3.2.3. Psychological distress

The 10-item form of the Hopkins Symptom Checklist-25 (HSCL; Derogatis, Lipman, Rickels, Uhlenhuth, & Covi, 1974) was used. The HSCL includes five items on anxiety and five on depression. Mean scores were calculated based on a 4-point response scale (‘not bothered at all’, ‘a little bothered’, ‘quite bothered’, ‘extremely bothered’). Cronbach’s alpha was 0.91.

#### 3.2.4. Impaired functioning

The participants answered five items adapted from the Utøya terror attack study (Hafstad, Thoresen, Wentzel-Larsen, Maercker, & Dyb, 2017) on how well-functioning they considered themselves to be in the following areas: (1) school/studies/work, (2) leisure time, (3) relationships with friends, (4) relationships with family, (5) home chores. The response categories were ‘functioning well’, ‘some problems’, and ‘many problems’. Mean scores were calculated. Cronbach’s alpha was 0.80.

#### 3.2.5. Social support barriers

The respondents answered five items regarding the degree to which they had refrained from seeking support or discussing their situation with others for the following possible reasons: (1) They are tired of hearing about it; (2) They have enough dealing with their own problems; (3) They would think I’m too caught up in it; (4) I don’t want to overburden my friends; or (5) Those who haven’t experienced it wouldn’t understand me. The scale was developed by the authors based on the Arnberg study (Arnberg, Hultman, Michel, & Lundin, 2012). Mean scores were calculated based on 5-point Likert-type scale scores, from ‘not at all’ (0) to ‘very much’ (4). Cronbach’s alpha was 0.84.

## 4. Data analyses

To investigate associations between exposure to severe childhood violence/bullying victimization and psychosocial adjustment, stepwise linear regression models were conducted. The psychosocial adjustment outcomes were regressed on severe childhood violence and bullying victimization, one at a time (Model I). Next, the two predictors were entered simultaneously, and the interactions between them were tested (Model II). Finally, adjustments for demographics were made (Model III).

Path analyses within the structural equation modeling (SEM) framework were conducted to examine the multivariate patterns of associations between the included variables. The mediational hypothesis was tested by using counterfactually based causal definitions of direct and indirect effects. The total effect of an independent variable on an outcome can be decomposed into two components: the pure natural direct effect (PNDE) and the total natural indirect effect (TNIE) (Muthén & Asparouhov, 2015; Pearl, 2014). The PNDE (direct effect) captures the effect of bullying victimization that is not accounted for by shame. The TNIE (indirect effect) captures the effect of bullying via shame. Causal mediation analysis relies on the untestable sequential ignorability assumption, which includes the assumption that there are no unmeasured confounders. In line with suggestions made by Muthén, Muthén, and Asparouhov (2016), we conducted sensitivity analyses that provide information regarding the robustness of our findings when this assumption is violated. The sensitivity analysis assumes various degrees of a correlation *ρ* between the error terms in the models for the mediator and the outcome, with zero correlation corresponding to sequential ignorability, and then calculates indirect effects for these conditions. The same sign for a large *ρ* interval indicates low sensitivity to violations of the sequential ignorability assumption. The analyses were conducted in SPSS version IBM, SPSS statistics versions 22 and 24, and Mplus 8.0 (Muthén & Muthén, 1998–2017). There was a small degree of missing data (missing items varied between 1–9). Valid percentages are presented.

## 5. Results

Of the total sample (*N* = 681), 24.5% (*n* = 167) had experienced bullying, and 7% (*n* = 48) had experienced severe violence. When these were combined into one variable, 21.5% (*n* = 144) had been exposed to bullying only, 3.7% (*n* = 25) to severe violence only, and 3.3% (*n* = 22) had experienced both. Of the individuals who had experienced bullying, 13.3% (*n* = 22) had also experienced severe violence, compared with 5% (*n* = 25) of the non-bullied individuals. Of the participants who had experienced severe violence, 46.8% had also experienced bullying, whereas only 23.1% of the non-violence-exposed individuals had experienced bullying (χ^2^ = 13.22, *df* = 1, *p* < .001).

### 5.1. Associations between bullying victimization, severe violence, and psychosocial adjustment

Experiencing bullying victimization and severe violence were independently associated with psychological distress, impaired functioning, and social barriers (see Table 1). The associations between bullying victimization and these psychosocial adjustment outcomes seemed to be stronger than the associations between severe violence and these outcomes. Interaction analyses showed that exposure to severe violence did not influence the relationship between bullying victimization and psychosocial adjustment. Adjusting for gender, age, financial situation, ethnicity, and parents’ mental health did not substantially change the main results, with the exception of the association between severe violence and social support barriers.

**Table 1.** Standardized estimates of associations between bullying victimization, exposure to severe violence, and psychosocial adjustment (*n* ~ 680), with results of main effects (Model 1), interaction between severe violence and bullying (Model II), and adjustment for gender, age, financial situation, ethnicity, and parents’ mental health (Model III).

	Psychological distress			Impaired functioning			Social support barriers		
	Model I	Model II	Model III	Model I	Model II	Model III	Model I	Model II	Model III
	Bivariate	Multi-variate	Multi-variate, adjusted	Bivariate	Multi-variate	Multi-variate, adjusted	Bivariate	Multi-variate	Multi-variate, adjusted
Bullying victimization	0.33***	0.32***	0.29***	0.31***	0.30**	0.28***	0.36***	0.34**	0.32***
Severe violence	0.17***	0.15***	0.13***	0.16***	0.12**	0.10*	0.16***	0.09*	0.06
Bullying victimization X Severe violence		−0.05	−0.06		−0.01	−0.02		0.04	0.05
Gender			0.20***			0.07			0.15***
Age			−0.05			0.01			0.04
Financial situation			0.11***			0.10**			0.09*
Ethnicity			−0.05			−0.01			0.04
Parents’ mental health problems			0.05			0.08*			0.04

** *p* < .01; *** *p* < .001.

### 5.2. The mediating effect of shame

**Figure 1.**
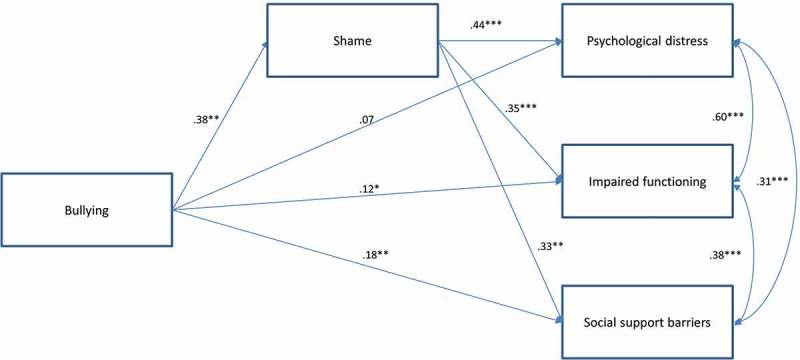
Overall path model. Standardized estimates of relationships between bullying victimization, shame, psychological distress, impaired functioning, and social support barriers. Adjusted for gender, age, financial situation, ethnicity, parents’ mental health problems, and exposure to severe violence.

We estimated a path model to explore the unique effect of bullying victimization and whether shame mediates the relationship between bullying victimization and psychosocial adjustment in young adulthood. The path model showed a direct relationships betweenbullying victimization, shame and the psychosocial adjustment outcomes, adjusting for demographics (see Figure 1). This model also accounted for the influence of exposure to severe violence.

To allow us to make inferences about the role of the mediator shame on the outcomes, we computed the PNDE and the TNIE (unstandardized) for each of the three outcomes. The effect of bullying victimization on the outcomes can be decomposed into two components: the effect on psychological distress that is not mediated via shame (PNDE) and the effect on psychological distress that is mediated via shame (TNIE). Again, we adjusted for severe violence, gender, age, financial situation, ethnicity (see Appendix B for the demographic estimates), and parents’ mental health. The TNIE was significant, estimate = 0.21 (95% CI 0.16–0.27, *p* < .001), indicating that shame partially explained why bullying victimization led to psychological distress. The PNDE was not significant, estimate = 0.09 (95% CI −0.01–0.19, *p* = .127). The PNDE and the TNIE were added together to reflect a total effect of bullying victimization on psychological distress (0.21 + 0.09 = 0.30). The ratio between the TNIE and the total effect (0.21/0.30) indicated that 70% of the total effect of bullying victimization on psychological distress was mediated via shame.

Next, we conducted the same analyses with impaired functioning as the outcome. The effect of bullying victimization on impaired functioning that is mediated via shame (TNIE) was significant (estimate = 0.12, 95% CI 0.08–0.16, *p* < .001), indicating that shame partially explained why bullying victimization led to impaired functioning. In addition, the PNDE was also significant (estimate = 0.10, 95% CI 0.03–0.18, *p* = .023), indicating an additional direct effect from bullying victimization to impaired functioning. The ratio between the TNIE and the total effect (0.12/(0.12 + 0.10)) indicated that 55% of the effect of bullying victimization on impaired functioning was mediated via shame.

Regarding social support barriers, we found that the effect of bullying victimization that was mediated via shame (TNIE) was significant (estimate = 0.21, 95% CI 0.16–0.29, *p* < .001), indicating that shame partially explained why bullying victimization led to social support barriers. In addition, the PNDE was also significant (estimate = 0.31, 95% CI 0.17–0.44, *p* < .001), indicating a direct effect of bullying victimization on social support barriers. The ratio of the TNIE to the total effect (0.21/(0.21 + 0.31)) indicated that 40% of the effect of bullying victimization on social support barriers was mediated via shame. The sensitivity analyses showed that the estimated indirect effects had the same sign as long as *ρ ≤* approximately 0.30–0.40, indicating moderate sensitivity to the assumption that there were no unmeasured confounders in these analyses. More specifically, the results of the analyses have a moderate probability of changing if unmeasured confounders occur.

## 6. Discussion

This study examined: (1) the psychosocial adjustment (distress, impaired functioning, social support barriers) in young adults who experienced bullying victimization and severe violence in childhood; (2) the unique effect of bullying victimization on psychosocial adjustment; and (3) whether shame mediated the relationship between bullying victimization and these outcomes in young adulthood.

### 6.1. Associations between bullying victimization, severe violence, and psychosocial adjustment

Our findings add to the growing literature documenting the detrimental long-term effects of bullying victimization. Our results show that experiencing bullying victimization and severe violence in childhood are associated with psychological distress, impaired functioning, and increased social support barriers in young adulthood compared with not experiencing bullying victimization or severe violence. Moreover, bullying victimization was uniquely associated with these negative adulthood outcomes even when accounting for the influence of severe violence. These findings emphasize that, although the bullying victimization may have ended, some victims may report poor mental health and struggle with adjusting to young adulthood in terms of problems managing daily chores, student life, work, and social life. This is consistent with previous studies documenting similar long-lasting effects (Sansone et al., 2013; Sigurdson et al., 2014; Strøm et al., 2013; Strøm, Thoresen, Wentzel-Larsen, Sagatun, & Dyb, 2014; Varhama & Björkqvist, 2005; Wolke et al., 2013).

Individuals who experienced childhood bullying victimization had higher odds of poorer outcomes compared with non-exposed individuals, and the co-occurrence with violence did not seem to worsen this association. This finding may imply that the consequences of experiencing bullying victimization are severe on their own and, thus, exposure to violence may not provide an additional risk. This finding was supported by the SEM analyses, which showed that bullying victimization had a unique effect on adult outcomes, despite accounting for exposure to violence.

Some children who are exposed to violence may develop impaired trust in others and may struggle to establish healthy social relationships (Bowlby, 1977; Killen, 2009). Some violence-exposed children may thus be particularly vulnerable to exclusion by peers or fitting in with a social group (Kim & Cicchetti, 2010). This could lead to risk for bullying victimization at school. In our study, we also observed a high degree of overlap between experiencing severe violence and bullying victimization in childhood. Approximately half of the individuals who experienced severe violence also experienced bullying victimization. This result is consistent with another recent study, which found that 40% of maltreated children had also experienced bullying (Lereya et al., 2015). The authors of that study suggest that previously documented effects of maltreatment, when studied apart from other forms of childhood victimization, may partly be due to experiencing bullying victimization. Finkelhor et al. (2007) argue that studying one form of childhood victimization in isolation may lead to an overestimation of its effects because of the large overlap among victimization experiences.

Our findings are aligned with a growing body of research acknowledging bullying as a form of victimization with severe, unique, and potentially long-lasting consequences, in contrast to previously held views that bullying is part of growing up and a harmless rite of passage (Olweus, 1997). The victims of bullying report a broad spectrum of psychosocial adjustment, including difficulty establishing social relationships, as shown in cross-sectional studies (Arseneault et al., 2010; Graham & Juvonen, 1998; Kim & Cicchetti, 2010; Olweus, 1993; Schacter et al., 2015). This study adds to our knowledge base by illustrating how childhood victims of bullying report problems with seeking social support in adulthood as they believe that they might be overburdening their friends with their problems, feel that others will not understand them, or believe that their friends have enough to deal with and are tired of hearing about their problems. This is in accordance with recent research documenting poorer social relations in adulthood for victims of bullying (Day et al., 2016; Sigurdson et al., 2014). The high correlation between social support barriers, impaired functioning, and psychological distress (shown in Figure 1) indicate that these factors are interrelated and that the consequences of bullying are non-specific and may impede multiple areas of functioning that potentially hinder successful adjustment to adulthood.

### 6.2. The mediating effect of shame

The mechanisms explaining the relationship between bullying victimization and psychosocial adjustment in young adulthood are important to understand. A significant contribution to the field is our finding that shame might mediate this relationship. Although bullying victimization had a direct and unique effect on the outcomes, its association with psychological distress was reduced to insignificance once shame was considered in the model. This was supported by the analyses showing that shame explained a large part of the association between experiencing bullying victimization and psychosocial adjustment in young adulthood. Shame has been associated with loneliness, social avoidance, and self-blame (Lutwak, Panish, & Ferrari, 2003; Nathanson, 1992; Rostami & Jowkar, 2016), indicating how it influence social interactions. During childhood and, especially, adolescence, involvement in one’s peer group becomes increasingly important. Being rejected and excluded from one’s primary social group may feel like a betrayal, which is one of the root causes of feeling shame. Thus, the processes of posttraumatic cognitive distortion after victimization plus the developmental vulnerability of adolescents to peer relationships may put the victims at a particularly high risk for shame.

Shame may result from taking on an ‘unwanted identity’ in the sense of being aware of appearing in an undesired way in front of an audience (Menesini et al., 2003). This may lead a person to want to hide her/his bullying experience and associated emotions, which may impede social support-seeking. However, as research on violence and shame has shown, this may lead to increased psychological distress over time. The scarce literature investigating this relationship supports that shame following bullying may lead to impaired mental health in adolescents, as it negatively affects cognitions, moods, and behaviours (Irwin et al., 2016). Considering the high correlation between psychological distress and impaired functioning, feelings of shame may affect this aspect as well. Taken together, our results illustrate that shame may be an important pathway to continue to explore in relation to bullying and its potential long-lasting negative effects.

### 6.3. Strengths and limitations

The strengths of this study include the use of behavioural-based questions regarding violence and bullying victimization, accounting for the influence of both bullying victimization and severe violence in the analyses, and examining multiple indicators of psychosocial adjustment. In addition, state-of-the-art mediation analyses were used.

However, there are some limitations. Although a community-based sample was used (T1), the majority of the respondents were ethnically Norwegian, which resulted in a less ethnically diverse sample. The SEM analyses only included individuals who had experienced a traumatic event because the shame items sought to measure trauma-related shame. Thus, the individuals in the reference group had all experienced a traumatic event (e.g. life-threatening disease; death of a loved one due to accident, murder, or suicide; serious injury; experiencing a terrifying situation). However, this may also be the case in ‘real-life’ reference groups, but in  study we accounted for this possibility as we specifically asked about previous traumatic experiences. As respondents may have reported multiple events, we do not know the experience to which the shame is related. However, this is not unusual when measuring other reactions to trauma, such as PTSD. Shame after one event is presumably not independent of shame after another event. In addition, we controlled for experiences of violence in the final analyses to focus only on bullying victimization. The samples for T2 and T3 were selected based on experiences with childhood violence; thus, the prevalence rates for bullying victimization are not representative of those of the general population. Attrition may also have resulted in a final sample that is not representative of the larger population. The timing of the measures may have influenced the results as bullying victimization, shame, and the three outcomes were measured at T3, while severe violence was measured at T1. The stronger association of bullying victimization than violence exposure with the outcome measures may be in part an artefact of the timing of measurement, with the four-year gap between the violence exposure assessment and the outcome measures possibly attenuating its relationship compared to the concurrent assessment of bullying and the outcome measures. Bullying mainly involves three roles (in addition to bystanders): the bully, the victim, and the bully victim (a bullying victim who also bullies others). A victim in our study could therefore have been either a victim or a bully victim. However, as we did not have adequate means of measuring the bullying of others, we unfortunately could not include bully victims (Olweus, 1997). Confounding factors that were not considered in this study, such as family support and family disadvantages, may have influenced our results. However, the sensitivity analyses only indicated moderate sensitivity to unmeasured confounders, which means that shame may still have a mediating role. Causal inferences cannot be made from these cross-sectional and retrospective data without replication with prospective longitudinal data

### 6.4. Implications for research and practice

Individuals experiencing bullying victimization in childhood are at a particularly high risk of poor psychosocial adjustment in young adulthood. Practitioners need to pay special attention to individuals who experience childhood bullying and be aware of the overlap with experiencing severe violence. Clinicians may want to screen for both. Clinicians need to be aware of shame as a response to victimization that may affect the individual’s mental health and social interactions. Shameful cognitions may be a potential area to target with therapeutic strategies. Indeed, treatment protocols may need refining to fully address the impact of shame on adaptation after severe violence and bullying victimization.

Research has shown that bullying victims are less likely to disclose their experience because of fears of peer disapproval, feeling weak/undermined, and preferring to maintain their autonomy (Boulton, Boulton, Down, Sanders, & Craddock, 2017). Thus, many victims will never get professional help. Efforts to reduce the possible feelings of shame following bullying victimization experiences could therefore be integrated into existing interventions, such universal school-based bullying prevention efforts. A study found that the second most important reason for disclosing bullying victimization (after stopping the bullying) was for the victim to feel better about him/herself (Boulton et al., 2017), which may correspond to reducing shame. Although efforts to prevent bullying are complex and the effectiveness of anti-bullying programmes have been discussed (Ferguson, Miguel, Kilburn, & Sanchez, 2007), efforts that engage bystanders by emphasizing that the bullying is not the victim’s fault (‘Kids who are bullied sometimes feel bad about themselves afterwards. This is not right! If you’re being bullied, it’s not YOUR fault!’) and to add shame as a programme outcome may be useful. Bullying is a group phenomenon and we know from previous research that bystanders play a crucial role in maintaining or limiting the level of bullying in school (Salmivalli, Poskiparta, Strohmeier, & Noam, 2012). Bystander intervention has also been useful for other areas of violence (Coker et al., 2014; Jouriles et al., 2015)

Research on the association between bullying victimization, shame, and psychosocial outcomes needs to be further explored. In particular, it would be interesting to include measures of shame in school-based surveys on bullying experiences that could be further utilized in prospective studies. This could expand our understanding of the relationship between bullying experiences of all types (e.g. bystander, aggressor, target, defender) and shame, which in turn would inform and improve current anti-bullying efforts.

## 7. Conclusion

This study contributes to the growing research documenting the long-term consequences of bullying victimization, reaching beyond health problems. The developmental phases of childhood and adolescence may create a particular vulnerability to shame after bullying victimization, which may influence psychosocial adjustment during the transition from adolescence to young adulthood. The unique effect of bullying victimization, over and above the effect of violence, calls for an integration of the two research fields to include bullying victimization and integrate it into trauma research, along with other forms of violence.

## Figures and Tables

**Figure A1.**
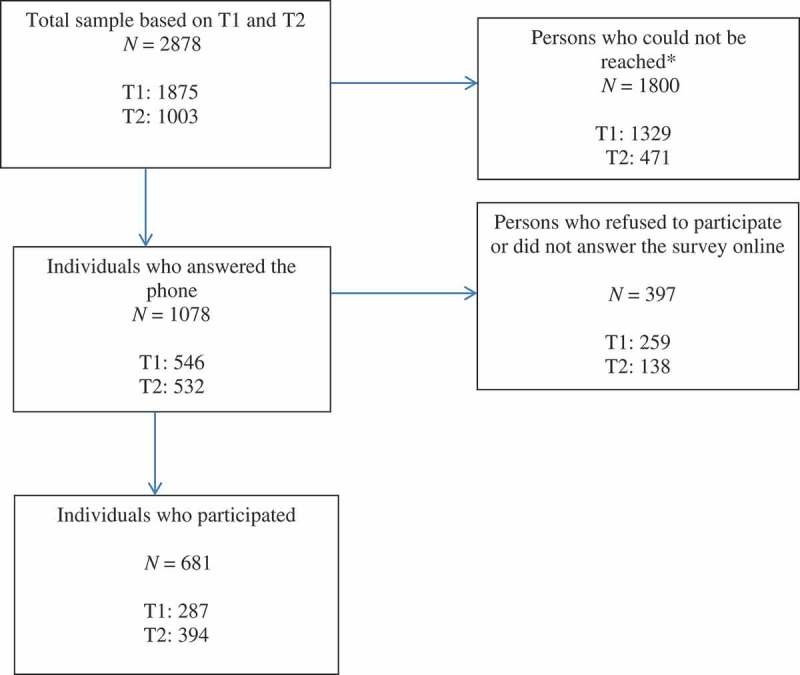
Flowchart for the follow-up study (T3). * Includes technical errors, no answer, incorrect registrations, incorrect numbers, and uncompleted follow-up appointments.

## Appendix B.

**Table A1.** Additional information on estimates of Figure 1: Standardized estimates of relationships between bullying, shame, psychological distress, impaired functioning, and social support barriers and the adjustment variables gender, age, financial situation, ethnicity, parents’ mental health problems, and exposure to severe violence. (n = 556)

	Bullying on	Shame on	Psychological distress on	Impaired functioning on	Social support barriers on
Gender	.08*	.12***	.14***	.02	.11**
Age	.00	−.13***	.01	.06	.04
Financial situation	.04	.14***	.04	.04	.04
Ethnicity	.02	−.01	−.04	−.01	.02
Parents’ mental health problems	.07	.01	.12**	.07	.08*
Exposure to severe violence	.10*	.18***	−.02	.00	.00

* *p* < .05, ** *p* < .01, *** *p* < .001
